# “Arsenic in Food”: Opinion Parading as Science

**DOI:** 10.1289/ehp.113-a225a

**Published:** 2005-04

**Authors:** Bruce K. Bernard

**Affiliations:** SRA International Inc. Washington, DC, E-mail: bernard@sra-intl.com

I write in response to apparent serious errors associated with Ellen K. Silbergeld’s letter in the May 2004 issue of *EHP* (Silbergeld 2004). As a toxicologist of nearly 30 years, a private consultant, and associate editor of the *International Journal of Toxicology*, I am concerned that although Silbergeld’s assertions on the risk of arsenic residues in poultry are presented under the cloak of good science, they appear to be her personal opinions and not a scientific conclusion based on sound methodology and evidence. In her letter I found at least three significant deviations from sound scientific methodology. These included the multiple mischaracterization of results presented in other publications and the introduction of a serious mathematical error. I will discuss in detail only one of these, the mathematical error, which should suffice to demonstrate the lack of science supporting Silbergeld’s opinion letter.

In one of the articles Silbergeld relied upon, “Mean Total Arsenic Concentrations in Chicken 1989–2000 and Estimated Exposures for Consumers of Chicken” by Lasky et al. (2004), the authors estimated that, based on the consumption of 60 g/day of chicken meat, an average individual may ingest 1.38–5.24 μg/day inorganic arsenic. However, in employing these numbers in her letter, Silbergeld stated the units erroneously and reported the results of Laskey et al. as 1.38–5.24 μg/kg/day inorganic arsenic. This single error inflated the alleged “exposure” rate by 7,000%, a significant miscalculation. In fact, this error, by itself, completely negates Silbergeld’s opinion that inorganic arsenic exposure through the consumption of chicken “would be a significant addition to drinking water exposure.”

This misquoting of Lasky et al.’s (2004) results is but one of Silbergeld’s significant mistakes in her letter. The result of each error of this type is either an inflation of the calculated exposure or a buttressing of Silbergeld’s stated opinion.

As a long-time author, reviewer, and editor of scientific papers, I am aware of the difficulty in ensuring the detailed accuracy of manuscripts, particularly where letters are concerned. There is a historical, although incorrect, perception that letters deserve less review than full manuscripts. At the same time some individuals, knowing that letters are not peer-reviewed to the same degree as scientific articles, make use of letters to get into print content that would otherwise not be acceptable. Although this may not have been the objective of Silbergeld’s letter, her scientifically unsupported opinion was repeated in the *Baltimore Sun* (O’Brien 2004) and other media (e.g., *Consumer Reports* 2005) as though it were scientifically proven fact. The result was unnecessary public alarm based on unsupported personal opinions.

Peer-review is meant to identify and weed out mistakes of this type. Ethical journals either require authors to correct errors before publication or decline to publish the article if the author refuses to make the warranted changes. Peer-review is not only the job of the publishing journal, but also the institution where the author resides (in this case, Johns Hopkins University). At many institutions, anything intended for publication must withstand internal review by an institutional committee before it can be sent to a potential publisher. For some reason, neither institutional nor editorial review detected these misquoted results and mathematical errors, a number of which appear to be obvious and would have been easily detected had the letter been checked. Both the journal and institution may wish to review their current procedures and make adjustments so they are not similarly embarrassed in the future.

As professionals, health scientists must be cognizant that respectability and trust are fragile commodities. We all know too well of a number of professions in our society that have lost significant amounts of respect and trust (e.g., politicians, lawyers, clergy) because of the misuse of the trust placed in them. Rational thought, balanced and unbiased evaluation, and honest reporting, as exemplified by the scientific method, are the primary underpinnings of the trust with which the nonscientific community honors us. Anything that causes loss of that trust, whether sloppy work or biased, self-serving presentations that distort the true state of scientific knowledge, demeans us all.
